# Osteoprotective effect of *Achyranthes bidentata* root extract on osteoporotic rats: a systematic review and meta-analysis

**DOI:** 10.1080/13880209.2024.2407531

**Published:** 2024-10-03

**Authors:** Hao Yang, Rui Tang, Hong-Li Wu, Jia-Hao Li, Chi Zhang

**Affiliations:** a The Clinical Medical College, Chengdu University of Chinese Medicine, Chengdu City, Sichuan Province, China; b The Health Preservation and Rehabilitation College, Chengdu University of Chinese Traditional Medicine, Chengdu City, Sichuan Province, China

**Keywords:** Phytomedicine, bone mineral density, bone biochemical markers, animal experiments

## Abstract

**Context:**

*Achyranthes bidentata* Blume (ABB), a plant of Amaranthaceae family, has been one of the more commonly used phytomedicine remedies for thousands of years, and recent studies have highlighted the efficacy of its extracts in the treatment of osteoporosis. Nonetheless, a thorough analysis of its benefits is currently absent.

**Objective:**

This meta-analysis assessed the effects of ABB root extract (ABBRE) on osteoporotic rats and provides a rationale for future clinical studies.

**Methods:**

Searches were conducted in seven different Chinese and English databases, and the search period was from their establishment to January 2024. This study was registered in PROSPERO (CRD42023418917). Selected research regarding the ABBRE treatment of osteoporotic rats, and the corresponding research has distinctly reported outcomes, and the data on the bone mineral density (BMD), bone histomorphometrics, biomechanical parameters, and bone biochemical markers of osteoporotic rats can be extracted.

**Results:**

Through screening, 11 studies met the eligibility requirements for inclusion, in which 222 animals were studied. The treatment group with ABBRE exhibited increased bone mineral density (standardized mean difference [SMD] = 1.64, 95% CI = 0.52 to 2.77). Based on subgroup analysis, the greatest increase in bone mineral density was observed when the dose of ABBRE was ≤ 400 mg/kg/day and the duration of treatment was ≤ 12 weeks.

**Conclusions:**

ABBRE is a phytomedicine that can effectively promote the enhancement of bone mineral density and ease osteoporosis. It can be developed into a new alternative therapy by conducting experiments and clinical studies on larger samples.

## Introduction

Osteoporosis, one of the rapidly growing diseases worldwide (Lane 2006), is marked by diminished bone mineral density and structural weakening of bone tissue throughout the body, resulting in heightened vulnerability to fractures and decreased bone strength (Arceo-Mendoza and Camacho 2021). Osteoporotic fractures, commonly referred to as fragility fractures, present substantial risks and stand as a primary cause of disability and mortality in the elderly population. Osteoporosis-related fractures have profound impacts on socioeconomic, societal, and psychological aspects (Liu et al. 2019). Fracture-related burden in Europe is projected to rise steadily, with estimated fractures increasing from 2.7 million in 2017 to 3.3 million in 2030, a 23% increase, accompanied by a 27% rise in annual costs €37.5 billion in 2017 (Borgström et al. 2020). Regarding the economic cost of osteoporosis treatment, the average direct cost per capita in Austria in 2019 is €151.8, compared to €104.8 in 2010, an increase of 45%, and Austria is ranked 6th in Europe in terms of the highest cost per capita for osteoporotic fractures (Willers et al. 2022). Osteoporosis in its early stages typically presents with no obvious clinical symptoms, making it difficult to detect. However, as the condition progresses, symptoms such as back muscle soreness, lower back pain, limb discomfort, fatigue, kyphosis or spinal deformity, height loss, and even fractures may gradually appear. Osteoporosis is an important public health problem that is often not recognized until the first fracture occurs. Major osteoporotic fractures (MOFs), or fragility fractures, are fractures caused by minor impacts such as a fall from standing height (Sànchez-Riera and Wilson 2017). Osteoporosis is three times more common in women than in men, and 71% of major osteoporotic fractures occur in women (Curry et al. 2018).

Currently, medications for osteoporosis treatment can prevent fragility fractures by inhibiting bone resorption and promoting bone formation (Yuan et al. 2019). It’s noteworthy that there’s an increasing concern about the potential side effects of these medications, including dizziness, leg cramps, jawbone necrosis, and an elevated risk of certain malignant tumours (Khan et al. 2015; Minisola et al. 2019; Li et al. 2020). Bisphosphonates (BPs), commonly used as anti-osteoporosis drugs, have side effects including acute phase reactions, which are the most prevalent short-term ones like fever, bone pain, nausea and vomiting, and an augmented risk of osteonecrosis of the jaw (Nasomyont et al. 2019). A dental examination ought to be conducted prior to the first utilization of BPs and if feasible, any invasive dental procedures should be accomplished before commencing the treatment with BPs (Rousseau and Retrouvey 2018). Teriparatide is commonly used for promoting bone formation but note that the Food and Drug Administration (FDA) believes continuous use beyond 2 years may cause toxic side effects like an increased risk of osteosarcoma (Gilsenan et al. 2020). Hence, there is a need to develop safer and less side-effect prone drugs for osteoporosis treatment. Traditional phytomedicine, with its extensive history, offers advantages such as holistic care, minimal side effects, and cost-effectiveness (Zhuo et al. 2022).

The compound formula with *Achyranthes bidentata* Blume (Amaranthaceae) is widely used in treating osteoporosis with remarkable results. Through randomized controlled trials in clinical practice, it was found that Bushen Huoxue Decoction can effectively relieve limb bone pain in patients with postmenopausal osteoporosis and improve lumbar spine bone mineral density. The effectiveness of the *Achyranthes bidentata* containing formula in osteoporosis prevention and treatment is established in clinical studies, but its modern pharmacological details are unclear, and it’s difficult to tell if it’s the single *Achyranthes bidentata* or a combination that works.

More than 270 metabolites like terpenoids, steroids, alkaloids, flavonoids, etc., have been isolated from *Achyranthes bidentata*. Recent research has extensively examined ABBRE’s effectiveness in osteoporosis treatment, showing its capacity to inhibit bone resorption, enhance bone formation, and bolster bone mass in laboratory and animal studies (Jiang et al. 2014; Suh et al. 2014; Yan et al. 2019).

A comprehensive review based on preclinical studies may contribute to providing conclusive evidence and offer valuable information for future experiments and clinical trials. This meta-analysis lays groundwork for forthcoming clinical trials exploring ABBRE’s therapeutic potential in osteoporosis management.

## Methods

The methodology of this study adheres to the preferred reporting items recommended by the guidelines for systematic reviews and meta-analyses (Liberati et al. 2009). The proposal utilizes the Sycle animal research tool and has been registered in PROSPERO (registration number: CRD42023418917) (Hooijmans et al. 2014).

### Literature search

The literature databases searched include PubMed, Web of Science, Foreign Medical Literature Retrieval Service, Embase, China National Knowledge Infrastructure (CNKI), Wanfang Data Knowledge Service Platform, and VIP Chinese Science and Technology Journal Database, covering both Chinese and English databases from inception to January 2024. We conducted searches using both keywords and Medical Subject Headings terms. From database inception to January 2024, we conducted searches using the following terms: (‘Osteoporosis’ OR ‘Osteoporoses’ OR ‘Post-Traumatic Osteoporoses’ OR ‘Post-Traumatic Osteoporosis’ OR ‘Senile Osteoporoses’ OR ‘Osteoporosis, Involutional’ OR ‘Senile Osteoporosis’ OR ‘Osteoporosis, Age-Related’ OR ‘Age-Related Bone Loss’ OR ‘Age-Related Osteoporosis’) AND (‘*Achyranthes bidentata*’ OR ‘*Achyranthes bidentata* Blume’) AND (‘Mice’ OR ‘Mouse’ OR ‘Rat’ OR ‘Rats’ OR ‘Animal’).

### Inclusion and exclusion criteria

The inclusion criteria for the study are presented as follows: (1) The study population is an animal model of osteoporosis; (2) The intervention drug is ABER, with the specified dose; (3) The control group is osteoporotic animals that are given the same dose of blank therapeutic fluid; (4) The outcomes include the effect of ABBRE on the bone mineral density, bone histomorphometry of osteoporotic animals, biomechanical parameters, and bone biochemical markers, and the original test should report one or more of the following outcomes: bone volume fraction (BV/TV); trabecular thickness; trabecular number; trabecular separation; fracture stress; maximum load; maximum stress; stiffness; SMI; serum osteocalcin (OCN); tartrate-resistant acid phosphatase (TRAP); serum alkaline phosphatase (ALP); C-terminal telopeptides of type I collagen (CTX); procollagen I aminoterminal propeptide (PINP); (5) Animal models are utilized for the study.

We examined the titles and abstracts of retrieved studies. The exclusion criteria are as follows: (1) Non-original and incomplete research articles; (2) Clinical trials, *in vitro* models, retrospective studies, case reports, and protocols; (3) Interventions that are different from ABER or do not have exact dosages and timings of administration; (4) The existence of concomitant interventions in the osteoporosis group. Further, full texts were assessed by authors, and publications without relevant outcomes were excluded.

### Selection of studies

After excluding duplicate reports, we independently assessed the titles and abstracts of the remaining articles to exclude ineligible studies. Full texts of the remaining articles that met the inclusion criteria were reviewed to determine their eligibility for inclusion. Discrepancies in the selections between the authors were resolved through mutual discussion.

### Data extraction

We independently assessed the data extraction of the included literature.

If there were any disagreements, the authors discussed and resolved them among themselves. The baseline characteristics of the study are as follows: method of inducing osteoporosis, sample size, effective substance, intervention measures, administration method, first author, publication year, study duration and the mean, standard deviation (SD) and standard error (SE) of the outcomes. SE was converted to SD using the formula (SD = SE × N^1/2^). In the case of multiple dosing, data were kept for the group receiving the highest dose. If data were presented graphically, GetData software was used to extract the necessary information.

### Quality assessment

We independently evaluated bias risk using the SYCLE tool, comprising ten items across six domains: (1) Selection bias, (2) Performance bias, (3) Detection bias, (4) Attrition bias, (5) Reporting bias, (6) Other bias. Studies meeting these criteria were considered low risk, while those not meeting them were deemed high risk. Studies with unclear bias descriptions were categorized as unclear risk. Any different opinions should be resolved through mutual discussions.

### Statistical analysis

The means and SD of continuous variables were recorded in Microsoft Excel. Review Manager 5.3.0 and Stata 16.0 were adopted for data analysis and visualization. Heterogeneity among the studies was evaluated using the heterogeneity index I^2^. When heterogeneity exceeds 50%, a fixed effects model is used; conversely, a random effects model is employed depending on the 50% threshold of heterogeneity. Subgroup and sensitivity analyses have been carried out to identify areas of variance, along with subgroup analyses by type of investigation. *p* < 0.05 was deemed statistically considerable. When I^2^ > 50%, subgroup analysis was performed to probe for sources of dissimilarity. Sensitivity analysis was carried out using the leave-one-out approach to determine the robustness of outcomes data. Publication bias was assessed using funnel plots and Egger’s test; *p*-values > 0.05 suggested that publication bias was absent.

## Results

### Retrieve results

**Figure 1.**
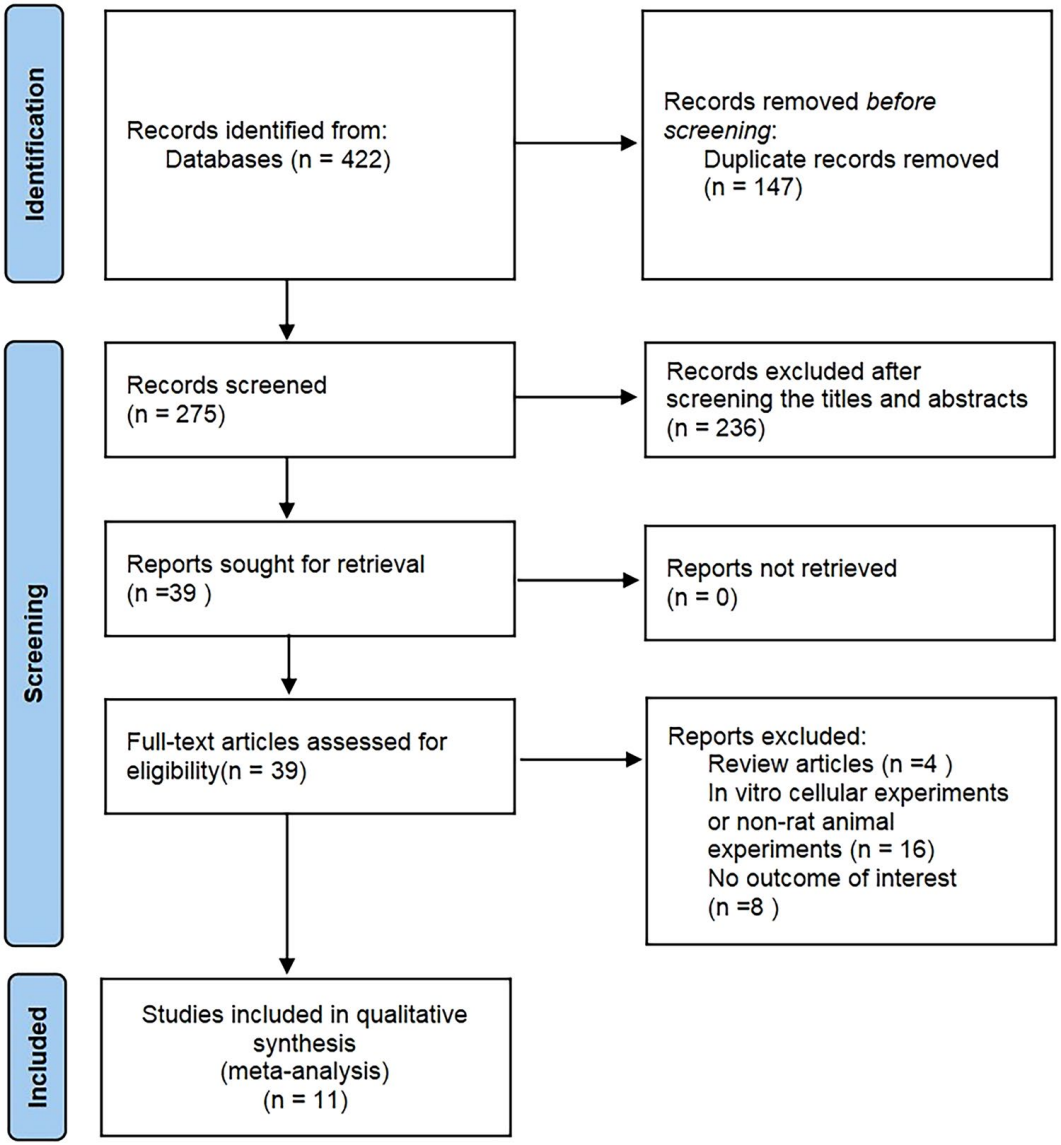
PRISMA flow chart of study selection. PRISMA, preferred reporting items for systematic reviews and meta-analysis.

After an initial search, a total of 422 articles were found. Subsequently, duplicate articles were excluded, as well as those whose titles and abstracts did not meet the inclusion criteria. The remaining 39 articles underwent full-text review. Among them, 25 articles were excluded because they were review articles, did not involve rodent experiments, or lacked valid data. Finally, 11 randomized controlled trials were included in this meta-analysis. The above selection process is shown in Figure 1.

### Characteristics of the study

This meta-analysis included 11 studies on the effects of ABBRE in improving osteoporosis in rats published between 2010 and 2024. All employed ovariectomized (OVX)-induced osteoporosis models, with a total of 11 cases. Regarding the isolation and purification of substances in ABBRE, there were seven articles concerning ‘ABB Polysaccharides’; one article about ‘N-butanol’; two articles on the isolation and extraction of ABB using ethanol, yet without specifying what the components were; and one article on the extraction with distilled water, also without specifying it. Rats in the experimental groups received oral ABBRE administration, with study durations ranging from 4 to 16 weeks. Table 1 outlines the key characteristics of the included studies.

**Table 1.** Characteristics of the included studies.

First author (year)	Induction of osteoporosis	Effective Substance	Sample size		Intervention		Methods of administration	Duration of study
			Treatment group	Control	Treatment group	Control		
He et al. 2010	OVX	*n*-Butanol	8	8	100 mg/kg/d	Equal normal saline	Intragastric	6 weeks
Lang et al. 2019	OVX	ABB Polysaccharides	20	20	400 mg/kg/d	Equal normal saline	Intragastric	3 months
Tao et al. 2019	OVX	Distilled water extract	4	4	75.6 g/kg/d	Equal normal saline	Intragastric	16 weeks
Wang et al. 2017	OVX	ABB Polysaccharides	10	10	10.4 g/kg/d	Saline 1 mL/kg/d	Intragastric	13 weeks
Yang et al. 2011	OVX	Ethanol extract	10	10	320 mg/kg/d	Equal normal saline	Intragastric	5 weeks
Yang et al. 2021	OVX	ABB Polysaccharides	10	10	8 g/kg/d	Equal normal saline	Intragastric	12 weeks
Yin et al. 2022	OVX	ABB Polysaccharides	4	4	1.08 g/kg/d	Equal normal saline	Intragastric	16 weeks
Zhang et al. 2012	OVX	Ethanol extract	10	20	500 mg/kg/d	Solvent vehicle	Intragastric	16 weeks
Zhang et al. 2018a	OVX	ABB Polysaccharides	10	10	400 mg/kg/d	Equal normal saline	Intragastric	13 weeks
Zhang et al. 2018b	OVX	ABB Polysaccharides	10	10	400 mg/kg/d	Saline 2 mL/kg/d	Intragastric	13 weeks
Zhang et al. 2019	OVX	ABB Polysaccharides	10	10	400 mg/kg/d	Equal normal saline	Intragastric	13 weeks

### Study quality

**Figure 2.**
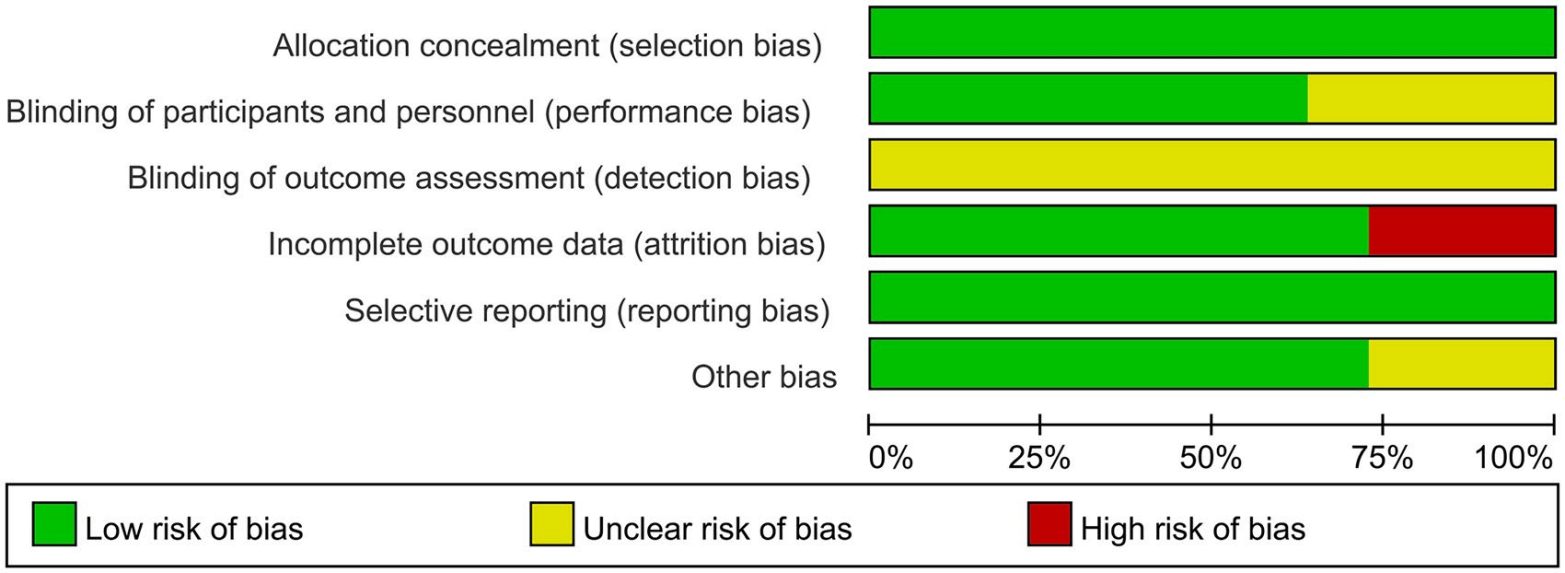
Quality of the included studies.

The bias risk assessment results for animal studies, as evaluated by the SYCLE tool, are summarized in Figure 2. The selection bias in Figure 2 consists of ‘random sequence generation’, ‘baseline characteristics’ and ‘allocation concealment’ respectively; the performance bias consists of ‘random housing’ and ‘blinding of trial caregivers’ respectively; the detection bias consists of ‘random outcome assessment’ and ‘blinding of outcome assessment’ respectively; the attrition bias consists of ‘incomplete outcome data’; the reporting bias consists of ‘selective outcome reporting’; and the other bias consists of ‘other sources of bias’.

None of the studies mentioned the following schemes: random sequence generation, allocation concealment, blinding of trial caregivers, random outcome assessment, and blinding of outcome assessment. The selection bias and reporting bias of all the studies were mentioned and were of low risk. The performance bias and other bias of some studies were unclear (36.4% and 27.3% respectively); regarding the attrition bias, three studies were of high risk (27.4%), and the rest of the studies were of low risk. Although the overall quality of the publications was not up to par, no literature was excluded based on its quality.

## Meta-analysis

### Bone mineral density

**Figure 3.**
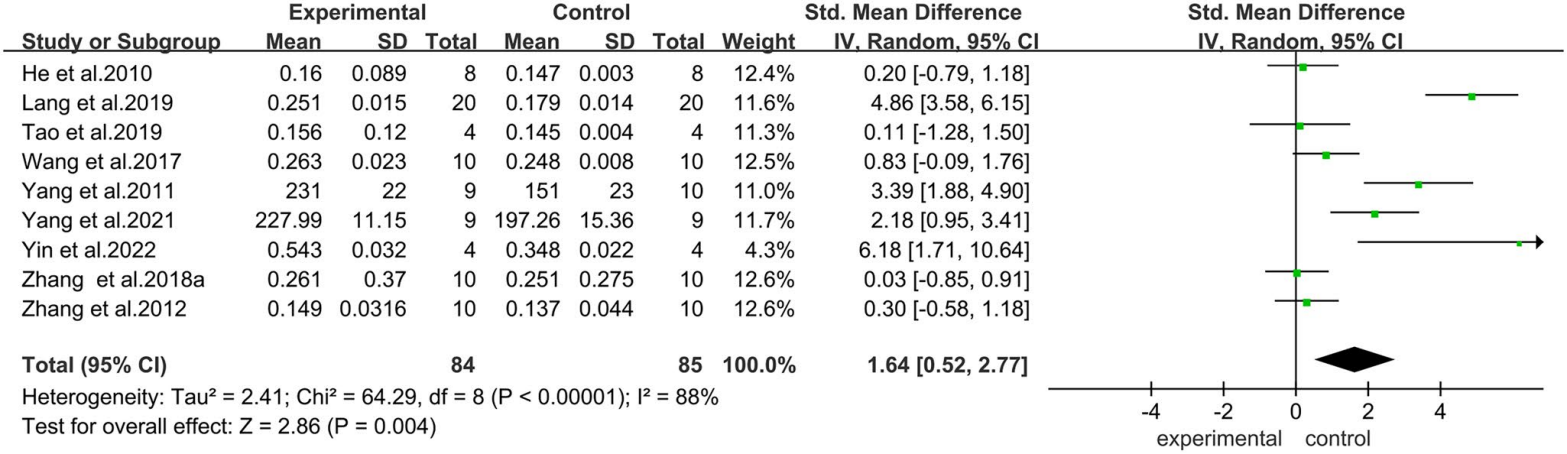
Forest plot comparing bone mineral density between the ABBRE group and the control group. SD: standard deviation; std: standard.

The analysis on ABBRE improving BMD in osteoporotic rats included nine studies and the results showed that the BMD in the ABBRE treatment group was significantly higher than that in the control group (SMD = 1.64, 95% CI = 0.52 to 2.77, *p* < 0.004) (Figure 3). Subgroup analysis indicated that different doses of ABBRE and duration of treatment led to a continuous increase in BMD (Table 2). The maximum increase in bone mineral density was observed when the dosage of ABBRE was ≤ 400 mg/kg/day and the treatment duration was ≤ 12 weeks.

**Table 2.** Subgroup analysis of bone mineral density according to the dose and duration of ABBRE treatment.

Subgroup	Standardized mean difference (95% confidence interval)	I²	*p* value
Dose			
≤400 mg /kg/d	1.80 [0.09, 3.51]	1.80 [0.09, 3.51]	0.04
>400 mg /kg/d	1.32 [−0.18, 2.82]	1.32 [−0.18, 2.82]	0.09
Duration			
≤12Weeks	2.63 [0.53, 4.73]	91%	0.01
>12Weeks	0.49 [−0.26, 1.24]	51%	0.2

### Bone histomorphometric and biomechanical parameters

**Figure 4.**
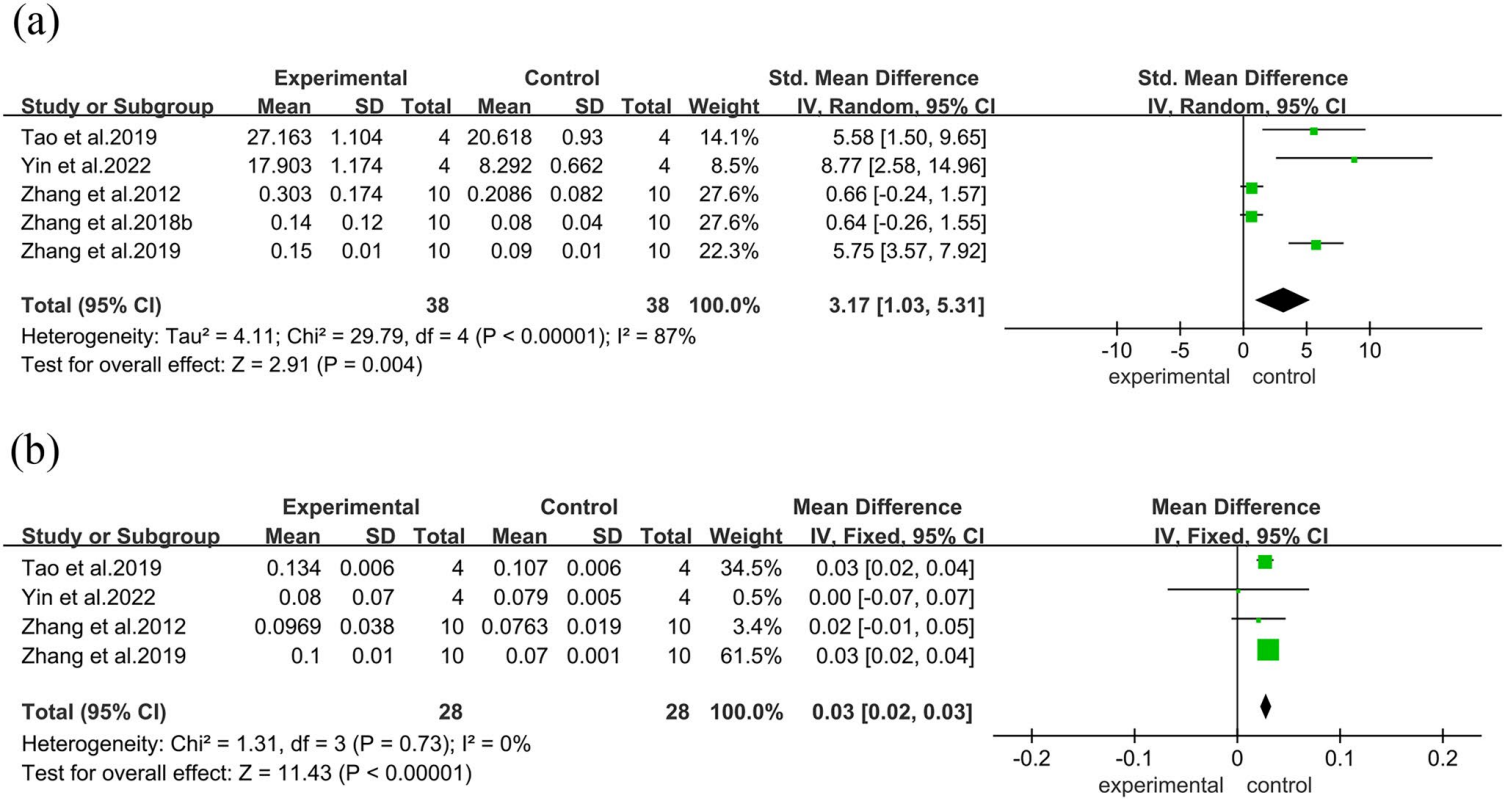
Forest plot between the ABBRE group and the control group. (a): BV/TV. (b): Trabecular thickness.

**Figure 5.**
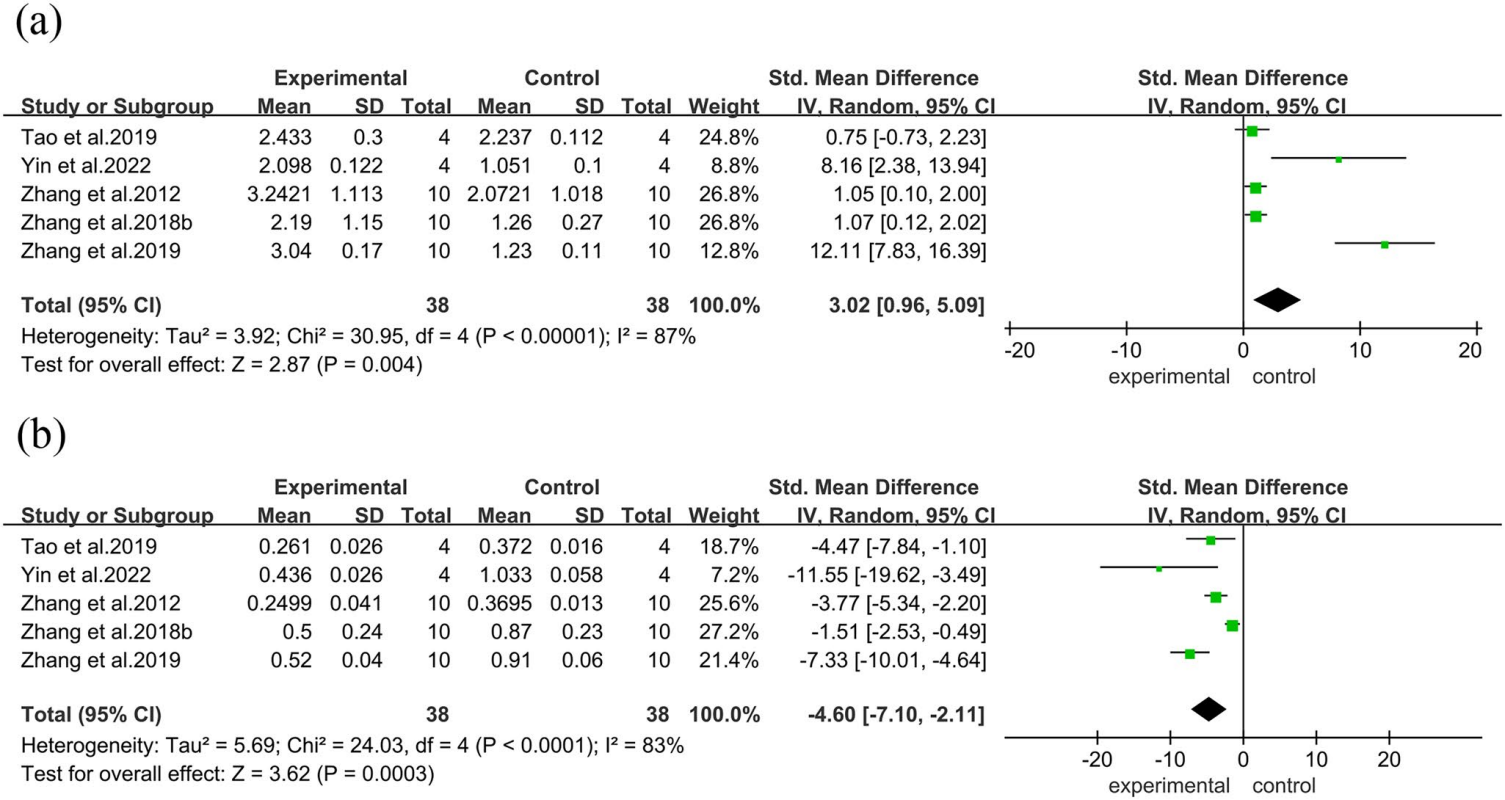
Forest plot. (a): Trabecular number. (b): Trabecular separation.

In the bone histomorphometry analysis, comprising five studies, ABBRE was found to increase BV/TV (SMD = 3.17, 95% CI = 1.03 to 5.31, *p* = 0.004). Four studies reported trabecular thickness (mean difference [MD] = 0.03, 95% CI = 0.02 to 0.03, *p* < 0.0001), (Figure 4) and five reported trabecular number (SMD = 12.11, 95% CI = 7.83 to 16.39, *p* = 0.004). Additionally, five studies demonstrated reduced trabecular separation with ABBRE treatment (SMD = −7.33, 95% CI = −10.01 to −4.64, *p* = 0.0003)(Figure 5).

**Figure 6.**
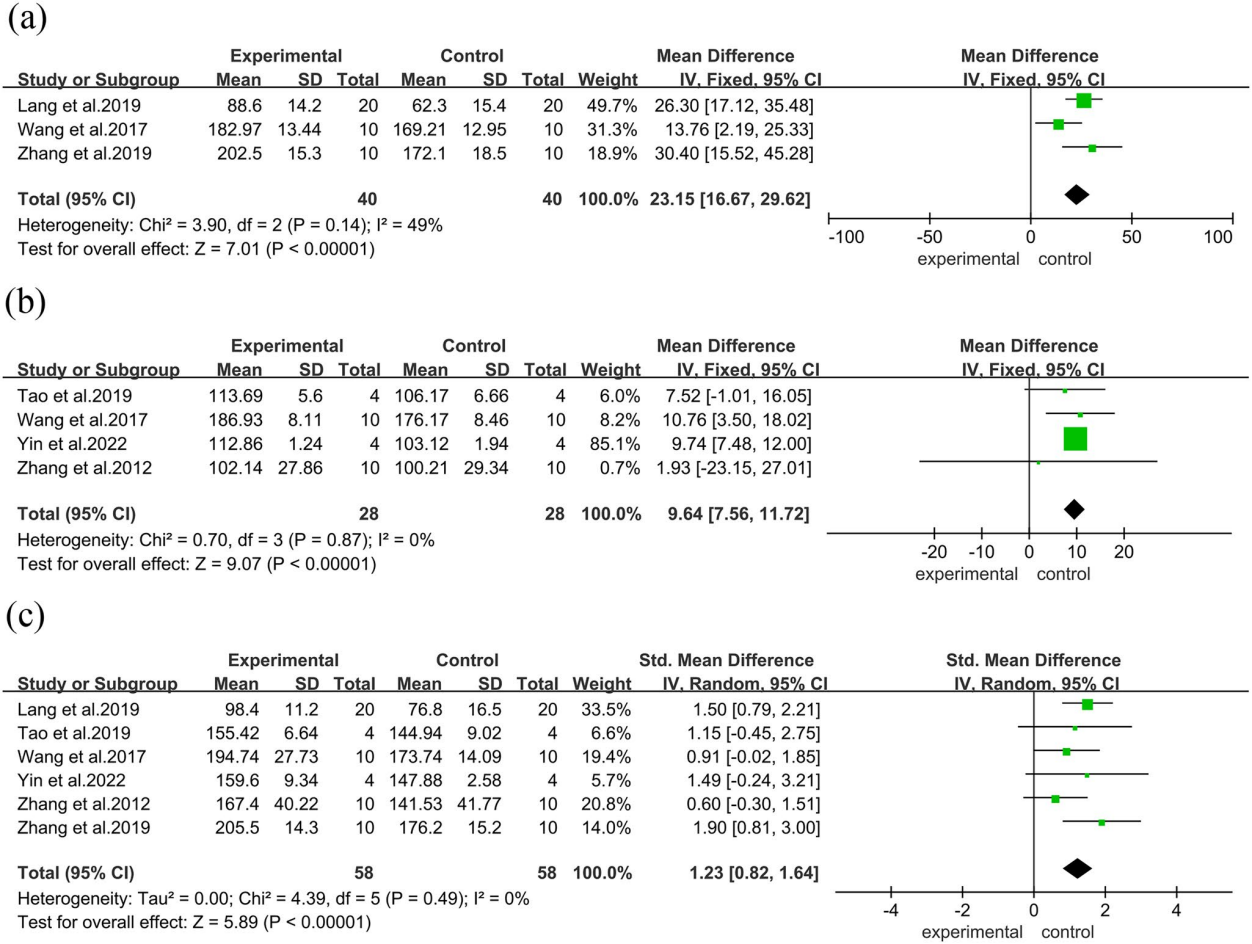
Forest plot. (a): Fracture stress. (b): Maximum load. (c): Maximum stresses.

**Figure 7.**
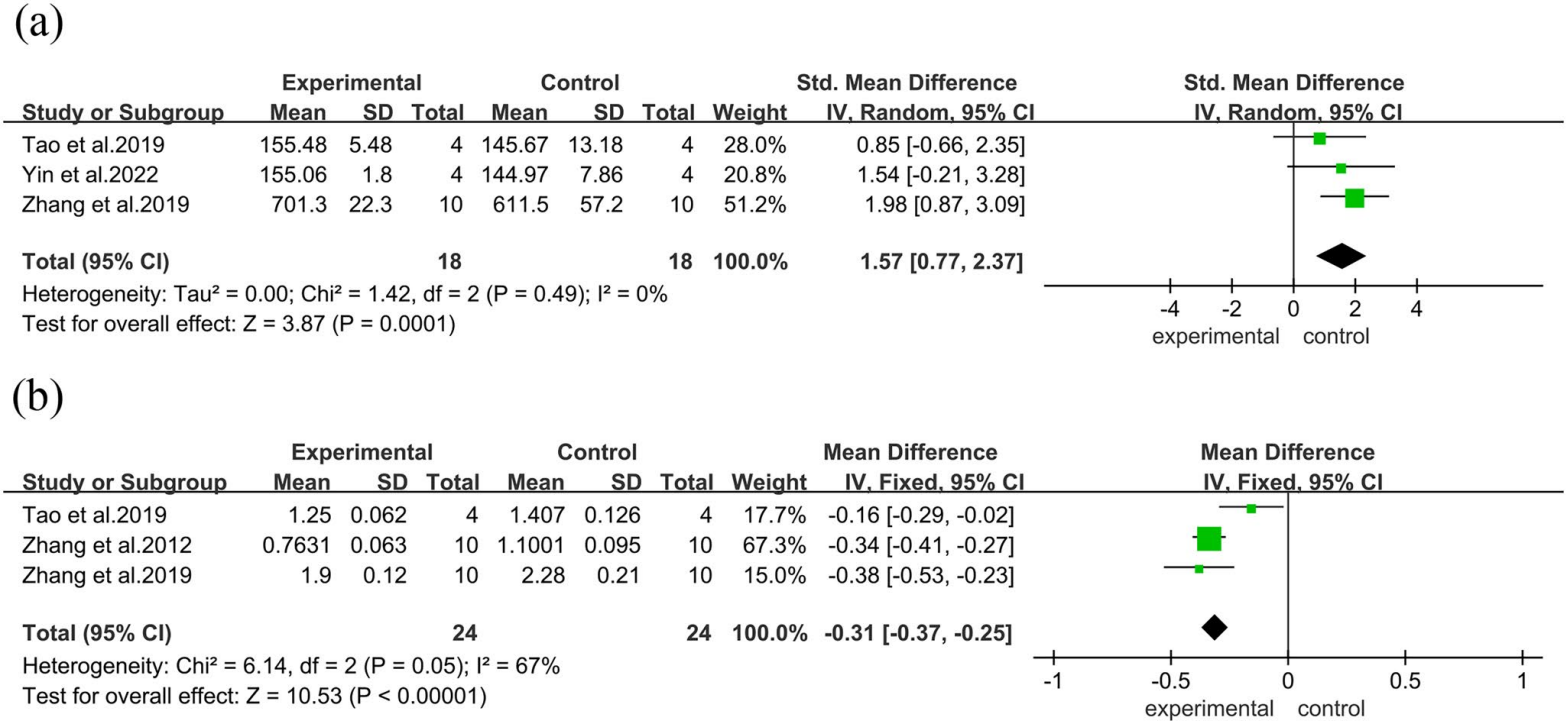
Forest plot. (a): Stiffness. (b): Structural model index.

Femoral midshaft specimens underwent three-point bending tests to assess bone biomechanical parameters. Additionally, in three studies, there was a substantial increase in fracture stress (MD = 30.40, 95% CI = 15.52 to 45.28, *p* < 0.00001). Furthermore, four studies reported a considerable increase in maximum load (MD = 1.93, 95% CI = −23.15 to 27.01, *p* < 0.00001). Six studies observed a substantial rise in maximum stresses (SMD = 1.90, 95% CI = 0.81 to 3.00, *p* < 0.00001), (Figure 6) along with a considerable increase in stiffness in three studies (SMD = 1.98, 95% CI = 0.87 to 3.09, *p* < 0.0001). Additionally, there was a substantial decrease in Structural Model Index (SMI) in three studies (MD = −0.38, 95% CI = −0.53 to −0.23, *p* < 0.00001)(Figure 7).

### Bone biochemical markers

**Figure 8.**
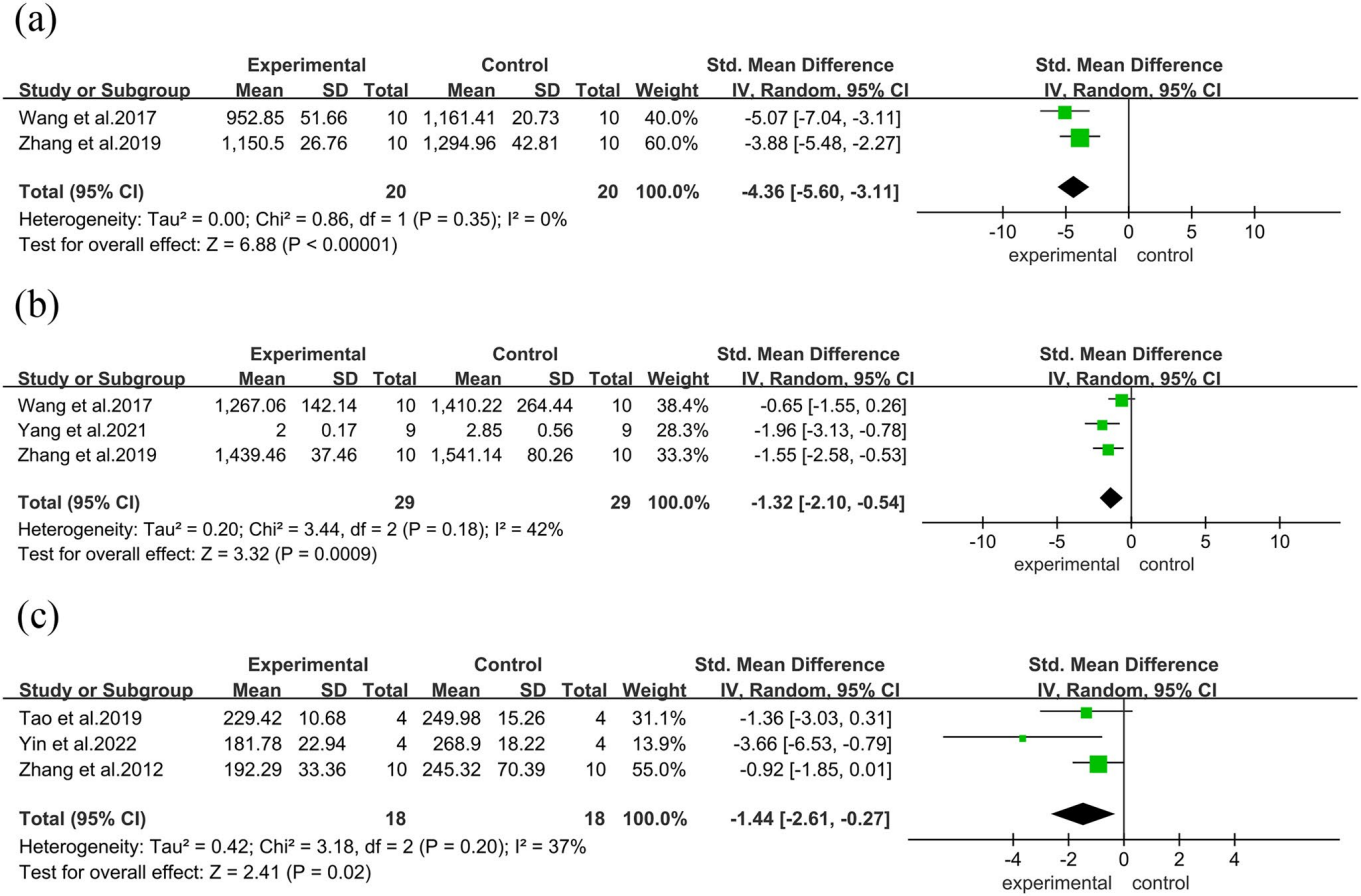
Forest plot. (a): Serum OCN. (b): Serum ALP. (c): TRAP.

**Figure 9.**
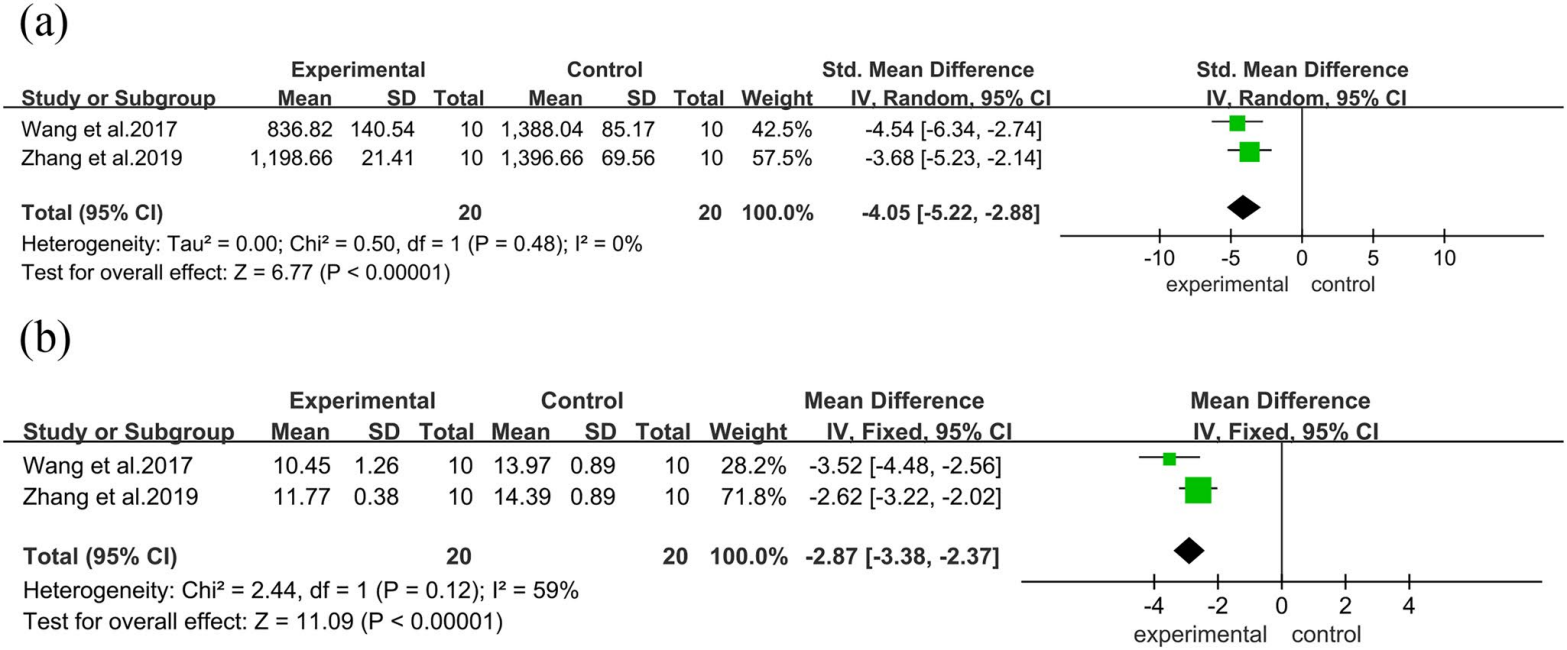
Forest plot. (a): CTX. (b): PINP.

In the analysis of serum in mice, a significant reduction in serum OCN was observed in two studies (SMD = −4.36, 95%CI = −5.60 to −3.11, *p* < 0.00001). Moreover, three studies reported a notable reduction in serum ALP levels in the ABBRE group compared to the control group (SMD = −1.44, 95%CI = −2.61 to −0.27, *p* = 0.02) and TRAP from two studies (SMD = −1.32, 95%CI = −2.10 to −0.54, *p* = 0.0009) (Figure 8). Furthermore, a significant decrease in CTX was observed in two studies (SMD = −4.05, 95%CI = −5.22 to −2.88, *p* < 0.00001), and in PINP in two studies (MD = −2.87, 95%CI = −3.38 to −2.37, *p* < 0.00001) (Figure 9).

### Sensitivity analysis and publication bias

**Figure 10.**
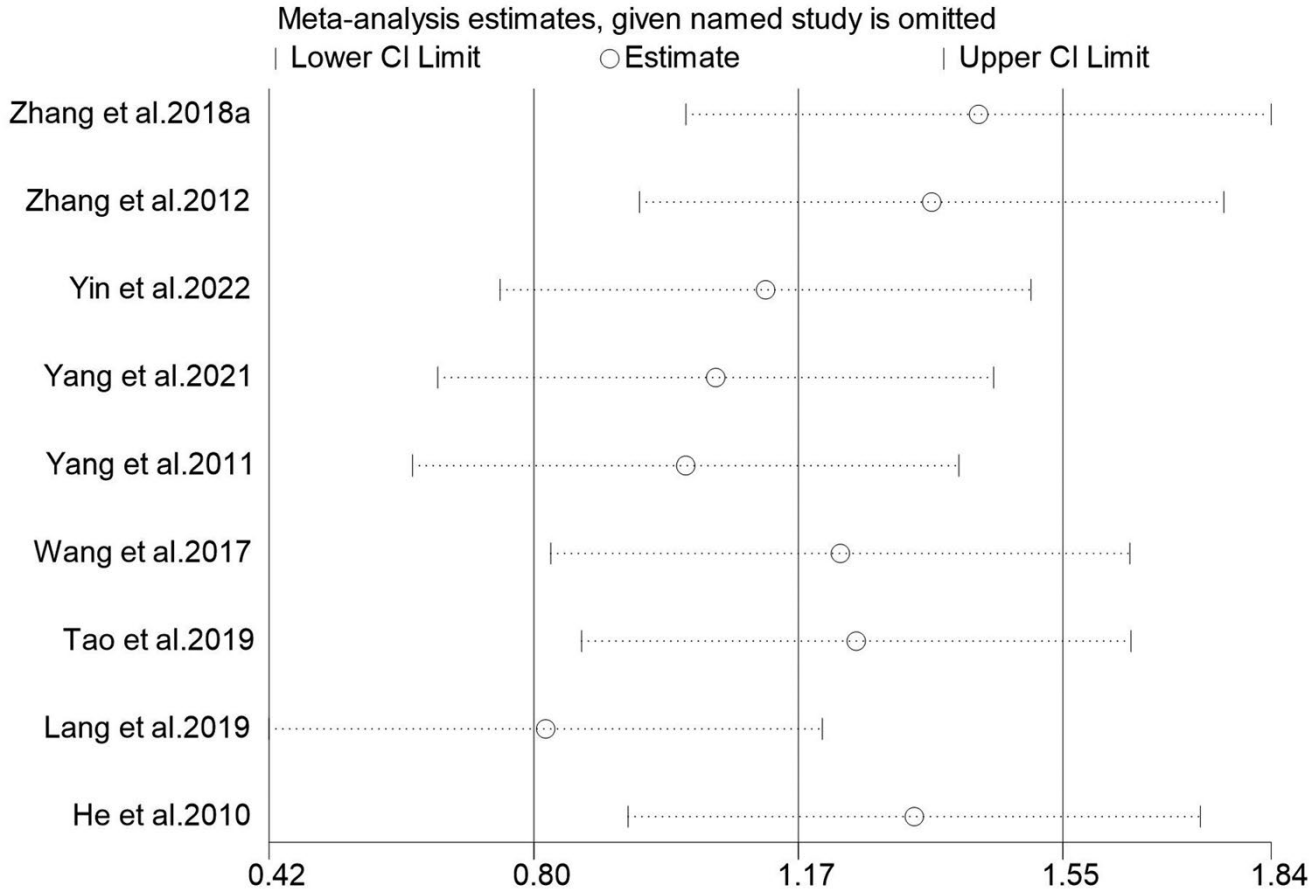
Sensitivity analysis of bone mineral density. CI: confidence interval.

Excluding any study with no significant changes in the heterogeneity index and 95% confidence intervals indicates minimal differences among the studies, affirming the robustness of the meta-analysis results (Figure 10).

According to the Cochrane Collaboration’s guidance, funnel plots and Egger’s test were excluded from the assessment of publication bias due to the relatively equal and small sample sizes of the included studies and the presentation of the outcome data as continuous variables.

## Discussion

This meta-analysis investigated the efficacy of ABBRE for the treatment of osteoporosis in a rat model. It showed a significant increase in bone mineral density and other indicators of bone strength in osteomalacia rats. In the results of the subgroup analyses, changes in the dose and duration of ABBRE treatment were essential factors in the association of increased BMD. The greatest enhancement in bone mineral density occurs when the dosage of ABBRE is ≤ 400 mg/kg/day and the treatment duration is ≤ 12 weeks.

Bone mineral density is considered the gold standard for diagnosing osteoporosis, and it correlates positively with bone strength (Fonseca et al. 2014). Osteoporosis characterized by low bone mineral density is a decrease in the quantity of bone unit size or per unit area, reflecting a decline in the number of bone tissues and bone substrates (Walker and Shane 2023). Bone histomorphometric analysis included symptoms such as BV/TV, trabecular thickness, trabecular number, and trabecular separation. In this case, bone volume is represented by the BV/TV, while the thickness and number of trabeculae show the variation in bone volume. Trabecular separation means the configuration of the trabeculae and is strongly correlated with bone volume. This meta-analysis suggests that cow knee polysaccharides have therapeutic potential for osteoporotic rats by improving the mentioned parameters.

Postmenopausal osteoporotic fractures and osteoporosis are common in clinical practice, and older women are at even higher risk, for whom hip fractures and spinal compression fractures are health destroyers. Through the study, it was found that lower oestrogen levels lead to decrease in bone tissue and microstructural degradation and ovariectomized female rats are severely oestrogen deficient, making them ideally suited to mimic postmenopausal osteoporosis (Khosla et al. 2011; Black and Rosen 2016).

There are many indicators for evaluating bone strength, among which skeletal biomechanical parameters are essential and intuitive (Díaz et al. 2016). The biomechanical testing of bone structural properties is a very important aspect in bone research. The examples of available biomechanical testing methods involve tensile strength testing, compression testing, torsional strength testing, as well as three-point and four-point bending and fracture testing (Mackert et al. 2023). Many studies employ mechanical testing to assess how bone structural properties are affected. Among these, the most essential basic structural properties are typically stiffness, strength, and toughness, as well as more complex properties such as fatigue resistance. Biomechanical tests assessing stiffness (usually SMI) and strength (evaluated based on the hardness or yield, ultimate, or fracture strength from tensile, compressive, bending, or shear tests) have a good track record (Bonfield 1987; Vashishth 2004). The results of this paper showed that ABBRE increased bone biomechanical parameters and had a role in promoting bone tissue strength in osteoporotic rats.

Bone undergoes continuous remodelling throughout life, with osteoblasts responsible for bone formation and osteoclasts regulating bone resorption (Saint-Pastou Terrier and Gasque 2017). Osteoclasts are multinucleated cells possessing a unique ability to resorb the bone matrix, and their excessive production or activation results in skeletal lesions and is a crucial cause for the development of osteoporosis (Veis and O’Brien 2023). When enhanced osteoclast formation and function disrupt the coordinated process of bone resorption and formation, bone resorption occurs. Research indicates that ABBRE, purified at different concentrations, does not markedly affect osteoblast proliferation. However, it does have a significant impact on the differentiation of primary osteoblasts at various concentrations (Yan et al. 2019). Different purification methods of ABBRE significantly boost MC3T3-E1 cell (Mouse calvaria-derived preosteoblast cell line MC3T3-E1) proliferation and elevate ALP activity over time. Moreover, the application of ABBRE upregulated the development of osteoblast-specific marker genes and proteins (Zhang et al. 2019). ABBRE inhibits osteoblastogenesis and bone resorption by suppressing the secretion of substances induced by receptor activators for nuclear factor-κB ligand (RANKL) (Song et al. 2018). The microbiota is an interesting target for alternative medicine interventions in osteoporosis, and it has been shown that ABBRE can modulate the development of osteoporosis through this pathway, the gut-bone axis (Yin et al. 2022). Of course, in numerous studies we can also see the limitations of the effect of ABBRE on osteoporosis, in some studies, it was found to have a significant osteoprotective effect on OVX rats and increase the differentiation of primary osteoblast cells, but it has little effect on the proliferation of primary osteoblast cells (Wang et al. 2017).

OCN, predominantly synthesized by osteoblasts, is the principal non-collagenous protein in bone. It involves orchestrating bone formation by aligning biological apatite (BAp) with collagen fibres, thereby influencing bone mass (Komori 2020). Serum PINP is a valuable indicator used to monitor the activity of Paget’s disease of bone, osteoblastic bone metastases, and osteoporosis treatment progress during follow-up (Koivula et al. 2012). Studies have reported that higher levels of serum CTX and serum TRAP are negatively correlated with BMD (Cao et al. 2012; Zhu et al. 2023). High serum ALP levels independently predict osteoporosis, especially in individuals undergoing androgen deprivation therapy, indicating a higher risk for this condition, particularly in those with lower body mass index (Hagiwara et al. 2022). This meta-analysis demonstrates that ABBRE effectively alleviates osteoporosis in rats by reducing the mentioned parameters.

## Strengths and limitations

This meta-analysis marks the inaugural comprehensive assessment of ABBRE treatment in osteoporotic rats, drawing from high-quality randomized controlled trials. Subgroup analyses delved into the effects of ABBRE dosage and treatment duration on bone mineral density. Nonetheless, methodological weaknesses and low-quality data in some studies, compounded by small sample sizes, limit the study. Future research should prioritize evaluating ABBRE effects in models mimicking postmenopausal and senile osteoporosis, such as ovariectomy and senile osteoporosis models.

## Conclusions

This meta-analysis reveals that ABBRE effectively enhances bone mineral density, improves bone parameters, and exhibits therapeutic potential for osteoporotic rats by analyzing alterations in bone biochemical markers. Based on the available technical data from this study, we conclude that ABBRE is a phytomedicine promising for promoting increased bone mineral density and improving osteoporosis. It can be developed into a new alternative therapy by conducting experiments and clinical studies on larger samples.
